# Disrupted Vessels—Connected Voices: Why Patient Partnership and Cross-Disease Collaboration Are Essential for Accelerating HHT Research

**DOI:** 10.3390/biomedicines13122997

**Published:** 2025-12-06

**Authors:** Irina Kruetzner, Freya Droege, Simone Kesten, Urban Geisthoff, Christian Hiepen

**Affiliations:** 1 Working Group Biomedicine and Cellular Biomechanics, Unit of Molecular Biology, Department of Engineering and Natural Sciences, Campus Recklinghausen, Westphalian University of Applied Sciences Gelsenkirchen, Bocholt, Recklinghausen, August-Schmidt-Ring 10, 45665 Recklinghausen, Germany; 2 Department of Otorhinolaryngology, Head and Neck Surgery and VASCERN HHT Reference Centre, Essen University Hospital, University Duisburg-Essen, Hufelandstr. 55, 45147 Essen, Germany; 3 Morbus Osler Selbsthilfe e.V., Scherenbergstr. 6, 10439 Berlin, Germany; 4 Department of Otorhinolaryngology, Head and Neck Surgery, University Hospital Giessen and Marburg, VASCERN HHT Reference Centre, Philipps Universität Marburg, Baldingerstraße, 35043 Marburg, Germany

**Keywords:** HHT, Morbus Osler, vascular malformations, endothelial cells, BMP, ALK1, endoglin, TGF-β, CCM

## Abstract

Rare vascular diseases such as hereditary haemorrhagic telangiectasia (HHT) represent a big challenge in biomedicine: complex pathomechanisms, limited patient material, and fragmented research communities slow down therapeutic progress. We argue that two elements are pivotal to bypass this problem. First, genuine partnership with patients—ranging from biospecimen donation to agenda setting—can unlock critical resources and align research with real-world needs. Second, molecular intersections between HHT and related pathologies call for coordinated, cross-disease programmes rather than isolated efforts. Recent multi-stakeholder gatherings hosted by patient organisations in Germany and elsewhere, such as the Second Scientific Symposium by the German HHT self-help group (Morbus Osler Selbsthilfe e.V.) in May 2025, have shown that when clinicians, basic scientists from different disciplines, and affected families co-design research questions, novel in vitro models can be generated more accurately, and pragmatic clinical trials emerge. Here, we outline actual opportunities for patient-integrated cellular model systems, shared biobanking, and comparative approaches across vascular malformation syndromes. In our opinion, letting informed and well-organised patient communities assemble such meetings opens unique opportunities twofold: on the one hand, the field can finally break out of its disease-specific silos; on the other hand, the development of novel HHT therapies could be accelerated by learning from progress in related pathologies.

## 1. Introduction

This article presents the authors’ perspective on advancing research in hereditary haemorrhagic telangiectasia (HHT) and related vascular disorders. It underscores patient partnership and cross-disease collaboration as essential for research progress, pointing out that these topics warrant greater focus within the field of rare diseases. The opinion is based on discussions during a recently held scientific symposium and on the current literature. This work is neither a systematic nor narrative review; it does not follow a systematic methodology. Furthermore, the authors would like to explicitly state that no financial support or promotional interests influenced the authors’ opinion. The aim is to provide an overview that reflects ongoing discussions and emerging opportunities within the complex field of rare vascular disorders, specifically HHT. To the best of our knowledge, no existing opinion article explicitly examines the role of cross-disease collaboration in rare vascular disorders or highlights how patient-organised scientific meetings can accelerate progress in this field. This article aims to address that gap.

## 2. Background: HHT

**Figure 1:**
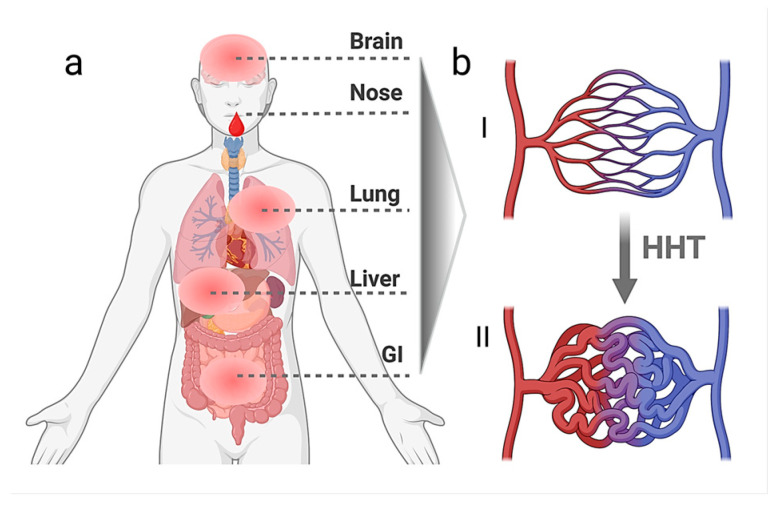
Most affected organs in HHT (**a**) with GI denoting the gastrointestinal compartment; healthy capillary bed (**bI**) and formation of vascular malformations (VMs) in HHT (**bII**)). Created in BioRender. Kruetzner, I. (2025) https://BioRender.com/lt2u7dg.

Hereditary haemorrhagic telangiectasia (HHT), also known as Rendu–Osler–Weber syndrome or Morbus Osler, is an autosomal-dominant, inherited disorder of the vascular connective tissue [1,2,3]. It is characterised by the formation of telangiectases and vascular malformations (VMs) in characteristic mucocutaneous sites and internal organs such as the liver, the lungs, the gastrointestinal organs, and the brain (Figure 1) [4]. To date, little is known about the complex molecular pathomechanisms causing this disease and the cell types that participate in the disease’s initiation and manifestation. The main genes identified thus far encode the cell surface receptors of the TGF-β (Transforming Growth Factor β) superfamily, the Bone Morphogenetic Protein (BMP) pathway co-receptor Endoglin (*ENG*) [5], BMP type I receptor ALK1 (*ACVRL1*) [6], and the downstream effector protein Smad4 [7].

Recurrent, spontaneous, and often severe epistaxis represents one of the earliest and most prominent clinical manifestations, typically accompanied by (muco-)cutaneous telangiectases. To this date, the Curaçao criteria are the consensus clinical diagnostic criteria for HHT, listed in Table 1. HHT is considered definite when three or four Curaçao criteria are met, possible with exactly two, and unlikely with one or none [8].

Chronic and acute complications in HHT can result from either arteriovenous shunting or bleeding. The rupture of cerebral VMs can cause haemorrhagic strokes, whereas rupture of nasal or intestinal lesions leads to recurrent epistaxis or gastrointestinal bleeding [4,9]. This often causes iron deficiency and anaemia, symptoms that require appropriate blood management and iron-replacement strategies to improve patients’ life quality [10].

When present in the lungs, pulmonary arteriovenous malformations (AVMs) create right-to-left shunting with impaired filtration function of lung vessels, causing brain abscesses and ischaemic strokes. As these complications are directly life-threatening, pulmonary screening is recommended for all individuals with a definite or possible HHT diagnosis [10,11].

VMs located in the liver—resulting in right-to-left hepatic shunting—can drastically increase cardiac output. Over time, this persistently hyperdynamic circulatory state may exceed the heart’s compensatory capacity, ultimately leading to high-output cardiac failure [12,13]. Furthermore, hepatoportal shunting can lead to portal hypertension, and portohepatic shunting might lead to encephalopathy [13,14]. In addition, all vascular shunts can cause blood steal, whereby surrounding tissues receive reduced oxygen supply as blood preferentially flows through the low-resistance channels of the VM.

To date, no causal therapy for HHT exists and treatment options for the individual symptoms are recommended in the Second International Guidelines for the Diagnosis and Management of HHT [10]. Hence, there is still much to learn about the molecular mechanisms and the clinical symptoms of HHT. Combining expertise from clinicians, various research fields, and patient’s experiences may help enhance understandings of HHT pathogenesis. These perspectives can be brought together and facilitated, for example, at patient meetings accompanied by a diverse set of scientific and medical stakeholders.

## 3. Rationale: The Added Value of Partnership

Scientific networks such as VASCERN, the European Reference Network for rare multisystemic vascular diseases, and the internationally active CureHHT organisation, serve as the overarching framework that connects and strengthens the global HHT community. They coordinate expertise, foster collaboration, and support research efforts across the HHT field.

Multi-stakeholder meetings like the Second Scientific Symposium hosted by the German HHT self-help group (Morbus Osler Selbsthilfe e.V.) in May 2025 reveal knowledge gaps on multiple sides. Clinicians, for instance, can explain acute bleeding management for newly diagnosed and unexperienced patients. They can demonstrate, e.g., nasal-packing options such as pneumatic balloons or resorbable tissue tamponades [15], discuss the risks of sclerotherapy, and outline the potential of oral therapy with tranexamic acid [10]. Veteran patients then add pragmatic tips—such as how to humidify airways, log epistaxis episodes, or handle fatigue at work. Molecular biologists complement both perspectives through patient-centred mapping signalling cascades that contribute to disease onset.

Inviting representatives from pharmaceutical companies to patient meetings offers the potential to recruit candidates for clinical trials and involves the patients directly in the development process. Integrating bioinformatics expertise—such as through Germany’s Medical Informatics Initiative (MII)—provides additional value. The MII unifies clinical data from all university hospitals in Germany, standardises symptom and diagnosis codes and aims to eliminate redundancies to expose disease patterns earlier. Patients shall gain faster diagnosis, timely treatment, and better quality of life, while the resulting datasets are made accessible for medical research [16].

The learning, however, is bidirectional: only patients can specify when epistaxis peaks (e.g., during nocturnal dryness or seasonal shifts) or how physical exertion might alter shear stress and force telangiectasia rupture. Such lived data feed back into, e.g., basic research in in vitro model development, computational simulation, and scalable platforms for drug screening purposes. This allows, e.g., the design and conceptualisation of experiments under more realistic conditions with the aim of recapitulating multiple environmental inputs essential for a better understanding of the complex pathomechanism of HHT.

## 4. Opportunities: Cross-Disease Synergies on Vascular Malformation Research

To investigate the molecular pathomechanisms of HHT in vitro and to facilitate novel drug screening approaches, adequate cell models must be established. As access to patient-derived primary vascular cells in rare diseases is limited and no central biobank for vascular anomalies yet exists, the technology of human induced pluripotent stem cells (hiPSCs) is receiving rising interest in HHT research. By comparing healthy and genetically modified hiPSC-derived vascular cells, it might be possible to uncover how BMP signalling imbalances contribute to HHT. During the Scientific Symposium, a previously established heterozygous HHT-2 hiPSC-line was presented [17]. These cells are currently used by two of the participating groups and provide a valuable contribution to HHT research.

Focusing on HHT genetics, the “second-hit hypothesis” was discussed, which suggests that a genetic mutation alone is not sufficient to trigger disease’s outbreak. Instead, an additional factor, such as a second somatic mutation in a previously healthy allele, might be necessary to initiate the development of VMs. This was already indicated by others [18] and in this context, the relevance of in vitro models with a second genetic modification was emphasised. The outcome of this debate was that HHT-specific cellular models containing such modifications should be established. It was also considered whether an additional “hit” might be conducted by altered blood flow or dysfunctional cellular biomechanics. Based on this hypothesis, hiPSC-models focusing on applying biomechanical stress to the differentiated endothelial cells are currently being developed by one of the participating research groups. Combined with microfluidics and other mechanical devices, those cells can be exposed in the laboratory to conditions that aim to closely mimic the human vasculature and physical influences on patients’ vessels.

Furthermore, an improved protocol for the scalable differentiation of hiPSCs into endothelial cells and for the generation of blood vessel organoids was presented by one research group [19]. This indicates ongoing efforts to establish sophisticated 3D cell culture models for rare vascular diseases. The group focuses on cerebral cavernous malformation (CCM), which leads to vascular abnormalities in the brain and is characterised by thin-walled blood vessels. With respect to these traits, CCM mirrors some aspects of HHT’s vascular phenotype. The learning from this was that integrating CCM-specific model systems and molecular targets into current HHT research strategies could unlock cross-disease synergies and raise the question of whether the pathomechanisms of other rare vascular disorders are more related to HHT than previously recognised.

Additionally, several BMP-related disorders, such as pulmonary arterial hypertension (PAH) and the rare musculoskeletal disorder fibrodysplasia ossificans progressiva (FOP), might also share signalling aberrations with HHT, although the causal mutations differ [20]. Nevertheless, there are some rare cases of the combined BMP-receptor type 2 and ALK1 mutation resulting in PAH and HHT within the same patient [21]. The imbalance between BMP and TGF-β signalling in PAH and FOP causes alterations in cellular biomechanics, changes in extracellular matrix composition, and the promotion of pathways leading to cellular plasticity; in both diseases, endothelial cells seem to undergo endothelial–mesenchymal transition (EndMT) [20,22,23]. During the meeting, it was discussed if there might be a similar pathomechanism in HHT. This would involve an overshooting of other TGF-β-related signalling pathways at the cost of BMP-9 signalling in endothelial cells, potentially also promoting EndMT and/or plasticity in HHT. Interestingly, also in CCM, the process of EndMT seems to play a crucial role during the development of vascular malformations [24]. Using adequate model systems will reveal whether similarities and common mechanisms between HHT, CCM, PAH, and FOP exist.

Beyond cells, interesting in vivo models increasingly enter HHT research. The zebrafish (*Danio rerio*) is an excellent in vivo model for studying vascular development and malformations. Its embryos are transparent, thus allowing researchers to directly manipulate and image vascular development in real time as the embryo grows. Using different gene-editing methods to replicate VM-associated mutations in zebrafish embryos allows for the analysis of the resulting vascular phenotypes. Here, the CCM field once again is a good example of how a seemingly distinct research area could enrich the HHT society through partnership since zebrafish are more commonly employed in CCM research. Therefore, the participants explored potential collaborations to develop novel zebrafish lines for HHT studies. These models would allow the observation of AVM formation in real time within a more complex and perfused 3D environment using live cell imaging.

By using genetically modified mouse models, it was previously discovered that AVMs in HHT originate in venous endothelial cells, rather than in the arterial system [25,26]. This finding marks a significant step forward in understanding HHT pathogenesis. During the session, participants underscored that endothelial cells in both HHT and CCM animal models exhibit similar abnormalities in the expression of Krüppel-like factor 2/4 (*KLF 2/4*). These transcription factors are key regulators of endothelial identity and vascular homeostasis, mediating responses to mechanical stimuli such as shear stress. *KLF* alterations were detected and relevant particularly in low-flow regions in CCM and high-flow areas of AVMs in HHT [27,28]. This suggests a potential link between KLF-related pathways in both diseases, despite the differing shear stress conditions present in the affected vascular abnormalities. Collaborative research on these pathways was discussed, as well as on tailored HHT animal models, with the aim of unravelling the detailed molecular mechanisms of HHT.

Having a look beyond model systems, the role of the immune system in HHT might be of special interest [29]. HHT itself is systemic and can manifest with a wide variety of symptoms and severity levels across patients. However, a reliable blood-based biomarker for HHT—ideally one that also tracks disease severity—has not been identified to date. In this context, a novel approach focusing on neutrophil granulocytes and their interaction with Pentraxin-3 (PTX3) was presented at the meeting. PTX3 is a soluble pattern recognition receptor secreted by various cell types, which plays an important role in immune defence by marking pathogens for elimination [30]. Interestingly, one participating group observed that PTX3 plasma levels appear to inversely correlate with haemoglobin levels in patients with HHT. Since severe cases often show lower haemoglobin levels due to frequent bleeding, this suggests that PTX3 might reflect disease severity. Nevertheless, further studies are needed to validate PTX3 as a robust and specific biomarker for HHT.

## 5. Conclusions and Future Directions

Patient gatherings unite affected individuals, clinicians, and researchers, aligning laboratory questions of improved biological model systems for drug testing with clinical needs. At the same time, patients and their families gain first-hand insight into molecular targets and realistic therapeutic timelines. This illustrates how face-to-face, community-driven encounters can strengthen motivation, mutual understanding, and, ultimately, translational progress.

For rare diseases like HHT, the lack of accessible and well-characterised biomaterials represents a major barrier to scientific progress. In this context, we support the creation of dedicated biobanks for diseases that lead to vascular disorders, in cooperation with medical focus centres, voluntary donors, and patient organisations. We also advocate for the development of a European-wide network to facilitate the collection and sharing of HHT samples. This initiative could significantly accelerate research efforts and support the discovery of new therapeutic strategies.

The meeting of the German HHT self-help group, together with participating researchers and clinicians, was a strong motivation to foster exchange between research fields that typically remain more separated but investigate shared and common pathomechanisms, model systems, and technical approaches. Now increasingly acknowledged, such patient-driven, transdisciplinary gatherings represent a valuable resource and can complement established scientific meetings. By promoting new dialogue, these events have the potential to accelerate progress in the HHT field.

The urge to create easily accessible biobanks and data repositories for vascular disorders, particularly rare disease patient material and data has become clear, along with the importance of better sharing and use of established and new model systems. In our opinion, instead of focusing on isolated research approaches, insights from disorders that are molecularly or phenotypically similar to HHT should be more intensely integrated, exploring the concept of cross-disease patterns of molecular dysregulation.

## Figures and Tables

**Table 1:** Curaçao criteria for HHT diagnosis, modified after [8].

Criteria	Description
Epistaxis	Spontaneous and recurrent nosebleeds
Telangiectases	At characteristic sites, multiple: the lips, fingers, oral cavity, and nose
Organ manifestation	Gastrointestinal telangiectasia; pulmonary, hepatic, spinal or cerebral vascular malformations
Family history	First-degree relative with HHT according to these criteria

## Data Availability

No new data were created or analyzed in this study.

## Abbreviations

The following abbreviations are used in this manuscript:

3D	Three-dimensional
ALK1	Activin Receptor-like Kinase 1
AVMs	Arteriovenous malformations
BMP	Bone morphogenetic protein
CCM	Cerebral cavernous malformation
ENG	Endoglin
EndMT	Endothelial-mesenchymal transition
FOP	Fibrodysplasia ossificans progressiva
GI	Gastrointestinal
HHT	Hereditary haemorrhagic telangiectasia
hiPSCs	Human induced pluripotent stem cells
KLF2/4	Krüppel-like factor 2/4
MII	Medical Informatics Initiative
PAH	Pulmonary arterial hypertension
PTX3	Pentraxin-3
TGF-β	Transforming growth factor β
VMs	Vascular malformations
